# Ultradeep Pyrosequencing of Hepatitis C Virus Hypervariable Region 1 in Quasispecies Analysis

**DOI:** 10.1155/2013/626083

**Published:** 2013-04-28

**Authors:** Kamila Caraballo Cortés, Osvaldo Zagordi, Tomasz Laskus, Rafał Płoski, Iwona Bukowska-Ośko, Agnieszka Pawełczyk, Hanna Berak, Marek Radkowski

**Affiliations:** ^1^Department of Immunopathology of Infectious and Parasitic Diseases, Medical University of Warsaw, 3c Pawińskiego Street, 02-106 Warsaw, Poland; ^2^Postgraduate School of Molecular Medicine, Żwirki i Wigury 61 Street, 02-091 Warsaw, Poland; ^3^Institute of Medical Virology, University of Zurich, Winterthurerstrasse, 190 8057 Zurich, Switzerland; ^4^Department of Medical Genetics, Medical University of Warsaw, 3c Pawińskiego Street, 02-106 Warsaw, Poland; ^5^Hospital for Infectious Diseases, 37 Wolska Street, 01-201 Warsaw, Poland

## Abstract

Genetic variability of hepatitis C virus (HCV) determines pathogenesis of infection, including viral persistence and resistance to treatment. The aim of the present study was to characterize HCV genetic heterogeneity within a hypervariable region 1 (HVR1) of a chronically infected patient by ultradeep 454 sequencing strategy. Three independent sequencing error correction methods were applied. First correction method (Method I) implemented cut-off for genetic variants present in less than 1%. In the second method (Method II), a condition to call a variant was bidirectional coverage of sequencing reads. Third method (Method III) used *Short Read Assembly into Haplotypes* (ShoRAH) program. After the application of these three different algorithms, HVR1 population consisted of 8, 40, and 186 genetic haplotypes. The most sensitive method was ShoRAH, allowing to reconstruct haplotypes constituting as little as 0.013% of the population. The most abundant genetic variant constituted only 10.5%. Seventeen haplotypes were present in a frequency above 1%, and there was wide dispersion of the population into very sparse haplotypes. Our results indicate that HCV HVR1 heterogeneity and *quasispecies* population structure may be reconstructed by ultradeep sequencing. However, credible analysis requires proper reconstruction methods, which would distinguish sequencing error from real variability *in vivo*.

## 1. Introduction

Genetic variability is a characteristic feature of hepatitis C virus (HCV), due to an absence of error correction mechanisms of the viral RNA-dependent RNA polymerase, fast replication, and recombination events [1–3]. As a consequence, HCV displays high intrahost population diversity, forming a pool of closely related but distinct genetic variants (*quasispecies*) [1]. The viral genetic variability is not evenly distributed through the entire genome; the highest variable regions include HVR1, HVR2, and HVR3 of the envelope E2 protein [4]. It is believed that HCV variability has significant clinical implications, since it may result in the generation of immune escape mutants, which may contribute to chronic infection and treatment resistance [5]. 

The detailed study of the minor variants within the *quasispecies *population is hampered by the absence of sensitive sequencing strategies which would allow for the detection of low-frequency genomes. The traditional method for studying viral *quasispecies* is based on Sanger sequencing of bacterially cloned viral sequences. However, this strategy requires extensive cloning to achieve the desired sensitivity for minor variants detection, a process, that is, costly and time consuming. Another limitation of the Sanger method is its difficulty in sequencing GC-rich regions. 

Other studies employed single strand conformational polymorphism (SSCP), an electrophoretic method shown to detect variants constituting as little as 3% of the viral population [6]. However, SSCP it is not informative of the nature of genetic changes or genetic distance between variants and therefore could not be used for some applications such as investigation of the drug resistance. In addition, in a mixture of heterogenous sequences, certain bands may overlap, underrating viral complexity. 

With next-generation sequencing (NGS) platforms, it is now possible to investigate viral *quasispecies* at much greater detail. Their high throughput allows for generation of millions of reads in a single sequencing run, facilitating in-depth sequencing. 

NGS can detect variants at low frequencies, which would go undetected by standard sequencing methods [7]. Nevertheless, in order to make reliable reconstruction of the viral *quasispecies* from the noisy, incomplete data obtained by NGS, a proper data analysis is required [8, 9].

In the present study we used ultradeep pyrosequencing (454/Roche) to characterize the complexity and heterogeneity of hypervariable region 1 (HVR1) in a patient persistently infected with HCV genotype 1b. This region was chosen as its protein product is under constant selection pressure of the host immune responses, especially of cytotoxic T cells and neutralizing antibodies [10, 11]. We sequenced this short region at very high coverage, aiming at detecting a large number of minority variants. We took into account sequencing errors in order to have a reliable reconstruction of the viral *quasispecies* on this region.

 Reports taking advantage of deep sequencing to investigate HCV genetic diversity for clinical and epidemiological studies are currently available [12–15]. Likewise, there are also works reporting and comparing bioinformatic approaches to infer the viral population from clinical samples, mostly HIV [9, 16–19]. Our study contributes progress in the evaluation of reconstruction methods and extends it for HCV *quasispecies *phenomenon investigation.

## 2. Patient and Methods

### 2.1. Sample

A serum sample from a 66-year-old treatment-naive female patient with genotype 1b chronic HCV infection was used. The serum HCV viral load was 1.54 × 10^6^ IU/mL. The patient provided informed consent and the study was approved by the Institutional Bioethical Committee. 

### 2.2. HVR1 Amplification

Viral RNA was extracted from 250 *μ*L of serum by a modified guanidinium thiocyanate-phenol/chlorophorm method using a commercially available Trizol reagent (Invitrogen) and suspended in 20 *μ*L of water. Five *μ*L of the solution containing RNA was subjected to reverse transcription at 37°C for 30 minutes using AccuScript High Fidelity Reverse Transcriptase (Stratagene). HVR1 sequences were amplified in a two-step PCR using FastStart High Fidelity Taq DNA Polymerase (Roche) as described previously [20]. Primers used for reverse transcription (E2 AS) and first round HCV HVR1 amplification (E2 S) were as follows: 5′-CATTGCAGTTCAGGGCCGTGCTA-3′ and 5′-GGTGCTCACTGGGGAGTCCT-3′. Primers for the second round PCR (E2 NS and E2 NAS) were as follows: 5′-CGT ATC GCC TCC CTC GCG CCA TCAG TCC ATG GTG GGG AAC TGG GC-3′ and 5′-CTA TGC GCC TTG CCA GCC CGC TCAG TGC CAA CTG CCA TTG GTG TT-3′. The latter contained tags recognized by GS Junior Sequencing System (underlined). 

### 2.3. SSCP Analysis of HVR1 Quasispecies

Second round PCR product was purified using Wizard SV Genomic DNA Purification System (Promega) and resuspended in 20 *μ*L of water. Next, 2–5 *μ*L of purified PCR product was subjected to SSCP assay as described previously [21]. Complexity of a population was reflected by the number of distinct bands. 

### 2.4. Ultradeep Pyrosequencing

Pyrosequencing was carried out according to the manufacturer's protocol for amplicons using GS Junior System (454/Roche). In order to lower contamination with short sequences (i.e., primer residues), HVR1 product of the second round PCR was purified from agarose gel by QIAquick Gel Extraction Kit (Qiagen). The extracted product was measured fluorometrically using Quant-iT PicoGreen dsDNA Assay Kit (Molecular Probes), and the amount of DNA equivalent to 3 × 10^7^ copies was subjected to emulsion PCR using GS Junior Titanium emPCR Kit (Lib-A). Pyrosequencing was performed according to the amplicon processing procedure for 100 cycles (recommended for amplicons up to 250 bp). 

### 2.5. Data Analysis

Reads that did not match primer sequences or had undetermined bases (Ns) were excluded from further analysis. Retained sequences of 179 bp were visualized using GS Amplicon Variant Analyzer (Roche). Subsequently, primer sequences were trimmed from the target sequence and reads of 138 bp were aligned to the reference sequence for genotype 1b HCV (GenBank accession number AJ406073) and translated to amino acid sequences by (*Molecular Evolutionary Genetics Analysis) *MEGA, version 5.0 (http://www.megasoftware.net/) [22]. Phylogenetic analyses were conducted in MEGA5 using the Maximum Likelihood method based on the Tamura-Nei model [23] using MEGA 5.0 software. Genetic parameters such as genetic diversity and sequence polymorphisms within sequences were 5 assessed by DNA SP version (http://www.ub.edu/dnasp/). The program diri_sampler from the ShoRAH software was used to correct sequencing errors and infer haplotypes. Given the high number of reads obtained in the sequencing, the dataset was split equally in two, and the obtained sets were analyzed independently. Error correction included mismatches as well as insertions and deletions.

## 3. Results

### 3.1. Amplification and Sequencing Errors

As our experiment used RT-PCR-amplified material, we attempted to assess the error rate in the consecutive experimental steps taking into account error rates of employed enzymes. For reverse transcription, AccuScript High Fidelity Reverse Transcriptase (Stratagene) was used, which displays three times higher fidelity than commonly used MMLV reverse transcriptase [24]. The estimated AccuScript RT error rate is 2 × 10^−5^ (manufacturers data). For PCR amplification, we used FastStart High Fidelity Taq DNA Polymerase (Roche), which has estimated error rate of 2 × 10^−6^ (three times lower than Taq DNA polymerase) [25]. Finally, the pyrosequencing error rate is estimated to be 1.07%, including mismatches (0.088%), insertions (0.541%), deletions (0.359%), and ambiguous base calls (0.085%) [26]. 

Studying clonal samples, or control samples where a set of clones are mixed in predetermined proportions are important to evaluate the error rate of the sequencing process and the performance of the haplotype reconstruction methods. Since these have already been reported elsewhere [16, 26], it seems not requisite to perform these experiments for every new study of the viral *quasispecies*.

### 3.2. Heterogeneity of HCV HVR1 Viral Variants Assessed by SCCP Analysis

**Figure 1:**
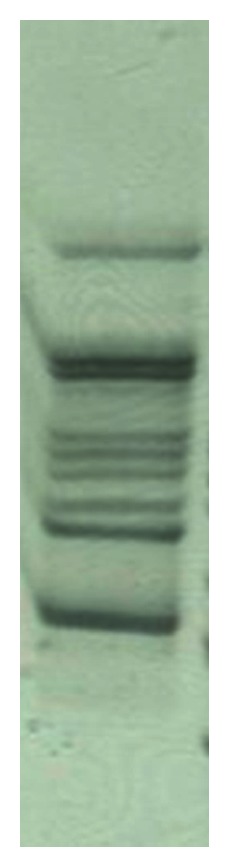
The SSCP image of HVR1 amplified from the serum of HCV-infected patient.

Based on gel analysis, at least nine SSCP bands of HVR1 were observed (Figure 1). The frequency was not uniform across variants, as could be seen by the different intensities of the bands.

#### 3.2.1. Heterogeneity of HCV HVR1 Assessed by Ultradeep Sequencing

To check the applicability of ultradeep pyrosequencing for HCV HVR1 heterogeneity analysis, the amplified product was sequenced by GS Junior System (454/Roche). Based on the data of GS Amplicon Variant Analyzer, the total number of sequenced nucleotides was 1.37 × 10^8^. The system read 76 332 individual sequences, among them 73 236 (95.9%) (28 098 forward and 45 138 reverse) were aligned to the reference sequence AJ406073 of genotype 1b HCV. The GS Amplicon Variant Analyzer software detected 15 917 haplotypes. Mean coverage of each variant (expressed by the number of identical reads) was 4.6. The most abundant haplotype coverage was 4540 reads. The rarest haplotypes comprised single sequence reads (74,6% of detected haplotypes). Our results are summarized in Table 1. 

#### 3.2.2. Error Correction in Haplotype Reconstruction

In order to reflect the HVR1 HCV population* in vivo *as accurately as possible, we explored the effect of different strategies to take the sequencing error rate into account. In a very conservative approach (Method I), we only considered variants detected at a frequency higher than 1%. This amounts to discard most variants, even if, given the high coverage, they appear in hundreds of reads. With this strategy we only retained 8 haplotypes.

A second strategy (Method II) consisted in requiring bidirectional coverage, that is, in only retaining variants supported by at least one forward and one reverse read. This method identified 40 HVR1 variants.

In the third approach we used the program diri_sampler from the software suite ShoRAH [16]. In this analysis, inference of the viral *quasispecies* is done in probabilistic manner using a Bayesian approach. It does not rely on the input of an error rate, rather, it estimates it from the sequencing data. Reads are clustered together and the consensus sequence of each cluster represents the original haplotype. Together with the frequency of each variant, the program enables assessments of the posterior probability of each haplotype, a confidence value for their existence. The number of diverse reads sequenced was higher than what the program can handle on a desktop computer with 4 GB of RAM. In order to face this limitation, we split the reads equally in two subsets, and performed haplotype reconstruction independently. Only haplotypes with confidence value >95% were retained. As an additional measure of reliability, only haplotypes supported by at least 5 reads were included. Since we are dealing with a coding sequence, frameshift inducing insertions/deletions were resolved correcting to the most common nucleotide for that position. As a result of two independent computations on raw data halves, two populations (A and B), consisting of 333 and 315 haplotypes respectively, were obtained. Their frequencies varied from 10.54% and 10.44% (the most abundant variants in population A and B, resp.) down to 0.013% and 0.014% (the least abundant variants in population A and B, resp.). 186 haplotypes were common to both populations and their frequencies were all above 0.02%. Seventeen haplotypes were present with a frequency >1%, constituting in total 58.6% of the entire population. 

#### 3.2.3. Characteristics of Inferred HVR1 Populations

After application of error correction methods, such parameters as percentage of mutated amino acid positions, genetic distance, genetic diversity as well as number of substitutions were calculated (Table 2). The highest genetic distance characterized population reconstructed by cut-off method (3.874) followed by ShoRAH method (0.110) and bi-directional coverage method (0.065), whereas genetic diversities were similar for all populations (0.923, 0.998 and 0.984 for method I, II and III, resp.). The highest number of nucleotide substitutions was detected in ShoRAH-reconstructed population (overall 70). 47 (67%) of them were present in genetic variants constituting more than 1% of the entire population.

**Figure 2:**
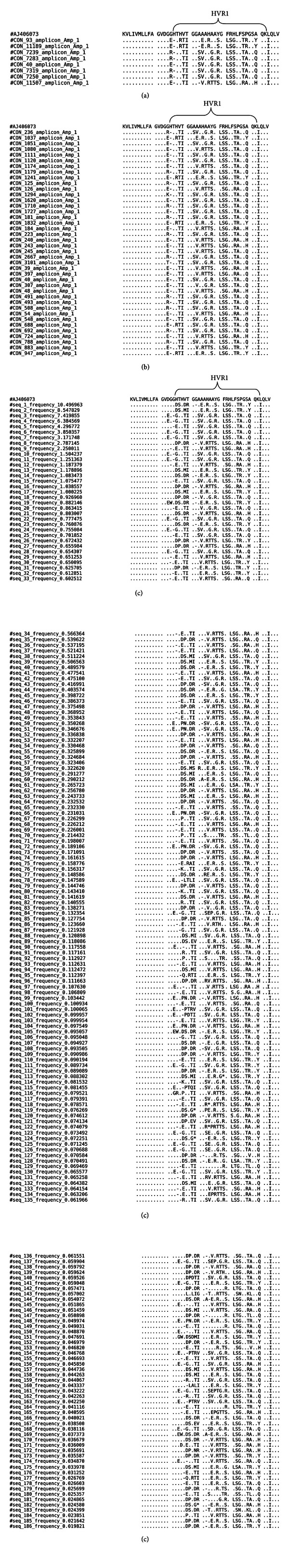
Amino acid sequences of HVR1 populations inferred after the application of three different error correction methods. (a) Cut-off method >1% (I), (b) bidirectional coverage (II), and (c) ShoRAH computation (III). Top sequence corresponds to reference sequence AJ406073 for genotype 1b HCV. Dots indicate consensus positions. Dashes indicate positions not present in the sequence. Asterisks indicate stop codons.

HVR1 populations were also compared on amino acid level (Figure 2). Within 27 amino acid stretch of HVR1, only 15 (55.5%) positions were polymorphic after application of methods I and II, and 20 (74.1%) after ShoRAH computations. Based on ShoRAH computation results, the most variable was the fourth HVR1 position, where 11 amino acid substitutions were detected when compared to reference sequence (V/D, V/M, V/T, V/L, V/R, V/A, V/E, V/G, V/N, V/I, V/Q). 

**Figure 3:**
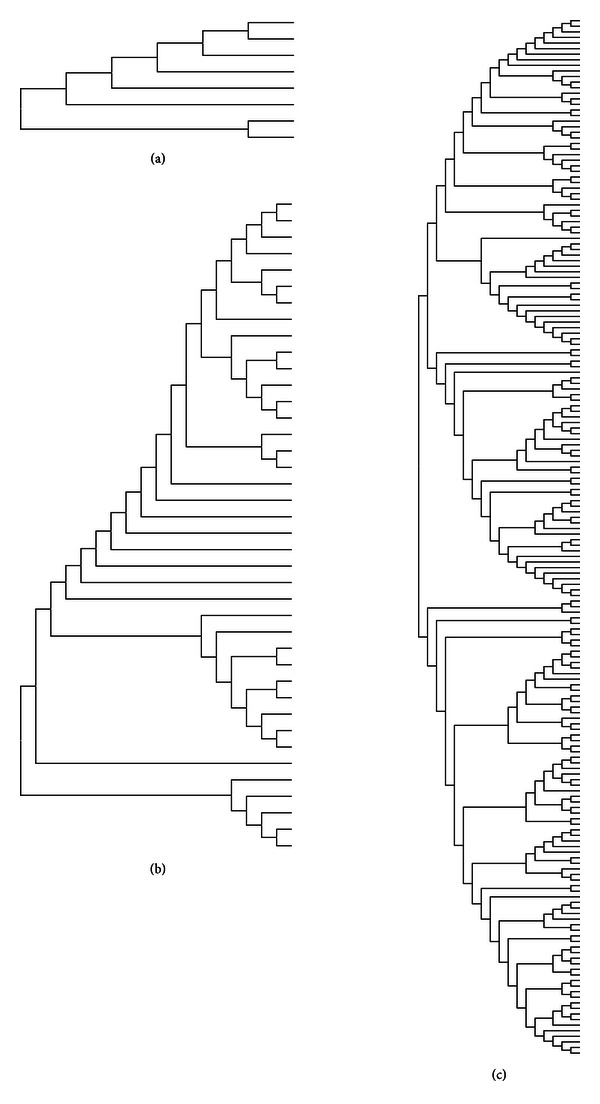
Molecular phylogenetic analysis of HVR1 populations inferred after the application of three different error correction methods. (a) Cut-off method >1% (I), (b) bidirectional coverage method (II), and (c) ShoRAH algorithm (III). The evolutionary history was inferred by using the Maximum Likelihood method based on the Tamura-Nei model [23]. Evolutionary analyses were conducted using MEGA 5.0 [22].

Viral populations were also analyzed phylogenetically. As shown in Figure 3, the general topology of three populations was similar. However, the tree topology based on ShoRAH computation was the most extensive.

## 4. Discussion

Pyrosequencing is a relatively novel technique which may help to decipher complex viral populations in terms of their diversity and structure. To date, it was successfully used in human immunodeficiency virus (HIV) research to identify minor drug resistant variants, analyze variable regions of heavy and light chains of neutralizing antibodies against HIV, as well as to determine HIV tropism, analyze superinfections and assess diversity of genital microbiota in HIV-infected women [27–31]. Ultradeep sequencing strategies also offers a new approach in HCV research. However, application of this method requires that several issues are taken into account. The foremost of these is the generation of mutations during reverse transcription and amplification reactions, due to enzyme errors [32]. Reverse transcriptase is the most error-prone, as it lacks a proofreading activity. For instance, error rate of common reverse transcriptases used *in vitro *to synthesize cDNA is at least 10^−4^ [24], and errors that occurred during this step are propagated during the subsequent PCR amplification. In the present study, in order to minimize errors, high fidelity enzymes were used in amplification reactions preceding sequencing (AccuScript High Fidelity Reverse Transcriptase and FastStart High Fidelity Taq DNA Polymerase). Nevertheless, the resulting hypothetical error rate of amplification is estimated to be lower than the sequencing error rate itself. The sequencing step introduces various types of errors related to the pyrosequencing chemistry and detection technology. The major contributor to errors is the ambiguity of homopolymer length, which results from the difficulty to resolve intensity of luminescence when a homopolymer is encountered. Moreover, insufficient flushing may lead to single base insertions. Overall, it was estimated that the mean error rate of pyrosequencing (defined as the number of errors such as miscalled bases or inserted or deleted bases divided by the total number of sequenced bases) was 1.07% [26]. This value may be considered as the experimentally confirmed resolution of the method. For the above reasons, the raw data obtained from sequencing should be additionally processed in order to remove low-quality reads and reads containing errors.

Three different error correction methods were applied to the raw sequencing data, which resulted in three HVR1 populations, differing in complexity and heterogeneity. The most sensitive was ShoRAH program reconstruction, which allowed to obtain the broadest spectrum of HVR1 sequences. This method has already been shown to reliably detect variants down to about 0.1% [33]. In this study we detected variants down to 0.02%, confirmed in two independent computations. The cut-off method, in which variants present in less than 1% of the population were discarded, was the least sensitive, as it allowed to detect only 8 haplotypes. Similar cut-off was applied in analysis of pyrosequencing reads of *pol/gag *of HIV population as well as *PePHD *E2 of HCV [14, 34]. It was reported that this method may result in inadequate haplotype reconstruction of low precision, low recall, or both, depending on the cut-off value. Too low cut-off value may result in low precision (fraction of true haplotypes among all called haplotypes) and conversely, high cut off may significantly lower recall (fraction of called haplotypes among all true haplotypes). For instance, based on the analysis of *gag/pol* HIV genes, it was shown that the cut-off of 50 read observations resulted in 80% precision but only 40% recall [33]. Application of the bi-directional coverage correction method II allowed us to determine the presence of 40 haplotypes, but such verification is laborious and raises concern regarding the acceptance of haplotypes characterized by high disproportion in forward and reverse strand counts. Among these forty sequences with bidirectional coverage, we could identify twenty that matched exactly one of the sequences obtained with ShoRAH. This is probably a very precise dataset, although, for example, some sequences might have a very biased forward/reverse read ratio and yet be included in it. Undoubtedly, using the strand information while reconstructing haplotypes is a strategy worth pursuing. A promising approach seems to be a proper statistical treatment of the strand bias, implemented together with the error correction of ShoRAH (McElroy, unpublished data).

In our study, using two independent ShoRAH computations, 186 haplotypes were reconstructed. In contrast, in the study of Bull et al. [13], 100 E2 variants were detected by ShoRAH. However, patients in that study were in an early phase of HCV infection and the study was performed along the whole genome, at much lower coverage, and thus HVR1 complexity could have been lower [13]. Based on ShoRAH reconstruction, we found that the most frequent HVR1 variants constitute a relatively small percentage of the entire population. Thus, the most abundant variant constituted 10.5%, and only 17 haplotypes were present in proportions higher than 1%. These data suggest that during the chronic phase of infection the *quasispecies *population is highly dispersed into minor variants, with no predominant sequences present. Similar haplotype frequency distribution was observed in foot-and-mouth virus population reconstructed by next generation genome sequencing [35]. In the only other HCV study investigating this issue, two to five variants were detected in frequency higher than 2.5%, whereas we detected eight such sequences. However, as already mentioned, viral samples in that study were drawn in the acute phase of infection [13]. The highest number of substitutions (70) within HVR1 was detected in population reconstructed by ShoRAH. Importantly, 47 (67%) of these were detected in variants constituting more than 1%. In contrast, during the acute phase of infection, less than 50% of substitutions were detected in variants present in more than 1% [13]. This suggests that, during the acute phase of infection, rare variants contribute more to the population diversity than during the chronic infection.

SSCP analysis, which has the sensitivity limit of 3%, revealed the presence of 9 bands. If the same cut-off value were applied in ShoRAH-based reconstruction, 7 haplotypes would have been identified. This fact suggests that the SSCP sensitivity might be higher than expected. 

However, it must be stressed that the absolute SSCP band number may not reflect the haplotype number as it represents resolved single DNA strands. Importantly, only sequencing provides information about the nucleotide sequence.

## 5. Conclusions

The newly available pyrosequencing technique opens a new approach to the analysis of complex viral genomes as it allows for detection of rare molecular variants. Better understanding of population genetics of complex viral populations seems crucial for understanding *quasispecies *phenomenon, viral evolution, and drug resistance. 

In the evaluation presented here, we used ShoRAH to obtain the broadest spectrum of HVR1 variants while trying to preserve their reliability. The use of different sequencing platforms, the optimization of library preparation, and data analysis will further improve the reconstruction of viral *quasispecies*.

## Figures and Tables

**Table 1:** HVR1 HCV characteristics obtained by pyrosequencing using GS Junior System (454/Roche).

Number of sequenced nucleotides	1.37 × 10^8^
Number of individual sequences that passed the quality control*	76 332
Number of individual sequences aligned to reference genome	73 236
Mean coverage per sequence	4.6
Identified haplotypes	15 917

*No undetermined bases, 100% match with primer sequences.

**Table 2:** The impact of haplotype reconstruction method on the variability parameters of HVR1.

Correction method	I Cut-off 1%	II Bidirectional coverage	III ShoRAH
Number of haplotypes	8	40	186
Number of nucleotide substitutions within HVR1	51	59	70
Percentage of mutated amino acid positions within HVR1 (%)	55.6	55.6	74.1
Genetic distance	3.874	0.065	0.110
Genetic diversity	0.923	0.998	0.984
